# Impact of equatorial Atlantic variability on ENSO predictive skill

**DOI:** 10.1038/s41467-021-21857-2

**Published:** 2021-03-12

**Authors:** Eleftheria Exarchou, Pablo Ortega, Belén Rodríguez-Fonseca, Teresa Losada, Irene Polo, Chloé Prodhomme

**Affiliations:** 1 Barcelona Supercomputing Center (BSC), Barcelona, Spain; 2 Departamento de Fisica de la Tierra y Astrofisica, Universidad Complutense de Madrid, Madrid, Spain; 3 Instituto de Geociencias, IGEO (CSIC-UCM), Madrid, Spain; 4 Group of Meteorology, Universitat de Barcelona (UB), Barcelona, Spain; 5 CNRM, Université de Toulouse, Météo France, CNRS, Toulouse, France

**Keywords:** Atmospheric dynamics, Ocean sciences

## Abstract

El Niño-Southern Oscillation (ENSO) is a key mode of climate variability with worldwide climate impacts. Recent studies have highlighted the impact of other tropical oceans on its variability. In particular, observations have demonstrated that summer Atlantic Niños (Niñas) favor the development of Pacific Niñas (Niños) the following winter, but it is unclear how well climate models capture this teleconnection and its role in defining the seasonal predictive skill of ENSO. Here we use an ensemble of seasonal forecast systems to demonstrate that a better representation of equatorial Atlantic variability in summer and its lagged teleconnection mechanism with the Pacific relates to enhanced predictive capacity of autumn/winter ENSO. An additional sensitivity study further shows that correcting SST variability in equatorial Atlantic improves different aspects of forecast skill in the Tropical Pacific, boosting ENSO skill. This study thus emphasizes that new efforts to improve the representation of equatorial Atlantic variability, a region with long standing systematic model biases, can foster predictive skill in the region, the Tropical Pacific and beyond, through the global impacts of ENSO.

## Abstract (web summary)

El Niño-Southern Oscillation (ENSO) is a key mode of climate variability with worldwide climate impacts. Here, the authors show that improved representation of summer equatorial Atlantic variability and its lagged teleconnection mechanism with the Pacific, relates to enhanced predictive capacity of autumn/winter ENSO.

## Introduction

El Niño-Southern Oscillation (ENSO) affects weather and climate throughout the world, from North and South America to Australia, India, Europe, and Africa^1^. By modifying the large-scale atmospheric circulation, ENSO-generated Tropical Pacific sea surface temperature (SST) anomalies are teleconnected to remote regions, affecting temperature and precipitation over land^2,3^ and impacting, as well the other ocean basins^4,5^. Interestingly, other basins like the Tropical Atlantic and Indian Oceans can also influence ENSO variability through changes in the atmospheric circulation^6,7^, both at multidecadal^8^ and interannual timescales^9,10^, and have been shown to contribute crucially to the development and predictability of ENSO^11^ and in particular for strong La Niña events^12,13^.

The ATL3 region in the East Atlantic (20°W–0, 3°S–3°N) is commonly used to characterize the equatorial Atlantic interannual variability. Analogous to ENSO variability and driven by similar mechanisms, ATL3 exhibits periodical warming/cooling events peaking in boreal summer, commonly referred to as Atlantic Niños/Niñas^14,15^. It has been shown that summer Atlantic Niños (Niñas) favor the development of Pacific Niñas (Niños) the following winter^9,10^. During an Atlantic Niño, anomalous heating induces wind convergence locally, altering the Walker cell by increasing upward motions on the Atlantic and subsidence over the Pacific. The latter leads to anomalous divergence at the surface that enhances easterly winds on the western Pacific. These in turn trigger an equatorial upwelling oceanic Kelvin wave that propagates eastward, cooling the surface and thus promoting the occurrence of a Pacific Niña event^9,10,16^. A positive Bjerknes feedback mechanism further maintains this ENSO phase^17,18^. Opposite changes occur during the Atlantic Niñas.

This teleconnection has been shown to be stronger after the 1970s^9,16,19^, likely related to the different background state of the Global Oceans, particularly on the Atlantic. Under a negative phase of the Atlantic multidecadal variability (AMV), the western equatorial Atlantic becomes warmer than normal, enhancing the ascending branch of the Walker cell, which is thought to create more favorable conditions for the Atlantic/Pacific teleconnection^19^.

A good representation of this teleconnection can be compromised in climate models because of the long-standing systematic biases in the Tropical regions^20^. These are particularly important in the Tropical Atlantic, where simulated climatological SSTs are generally warmer than in observations by several degrees^21^, presenting also important differences in their spatial distribution^22^. Several physical reasons have been proposed to explain those biases, such as too weak equatorial winds, excessive precipitation, too few stratocumulus clouds and excessive solar radiation, as well as insufficient oceanic mesoscale eddy advection, and insufficient oceanic upwelling^23–25^.

Among the major consequences of the mean state Tropical Atlantic biases, recent studies suggest that they can lead to a poor representation of the local SST seasonal cycle^26^ and through it of the simulated interannual variability^27^, affecting also the Tropical Pacific SST seasonal cycle and mean state^28^. Tropical Atlantic biases can also potentially weaken the Atlantic–Pacific teleconnection^29^, and deteriorate the representation of the Tropical Pacific climate^30^. However, no clear consensus has been reached on whether SST biases and variability of the Tropical Atlantic at large can also influence the prediction skill of ENSO^11,25,26,31,32^.

## Results

### Assessment of the teleconnection in a multi-model ensemble

In this study, we assess in a multi-model framework the link between the prediction skill of the Atlantic and Pacific Niños, and whether this is sensitive to the representation of the Tropical Atlantic–Pacific teleconnection. For this we combine our own forecast system, based on EC-Earth, with a selection of forecasts from the multi-model ensembles NMME and EUROSIP, to create an ensemble of 15 systems initialized in June, that is when the teleconnection starts developing (see “Methods”). Another advantage of using June initialization is that the Atlantic variability and predictability in the forecasts is decoupled from the previous winter ENSO event, allowing us to focus exclusively on the Atlantic–Pacific teleconnection pathway.

**Fig. 1:**
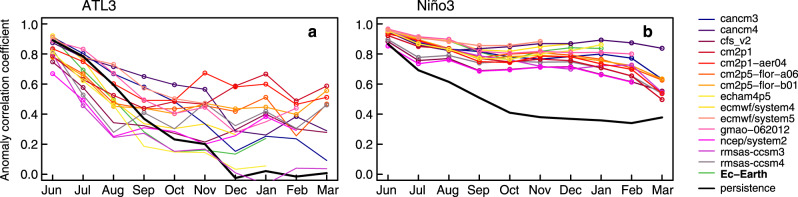
Multi-model assessment of seasonal prediction skill in the Tropical Atlantic and Pacific. **a** Prediction skill (evaluated by the anomaly correlation coefficient; ACC) of ATL3 SST in each of the prediction systems in Supplementary Table 1. Skill is evaluated against HadISST v1.1, for the period 1981–2011. ACC values associated with persistence in observations are indicated by the black line. **b** The same but for the prediction skill of Niño3 SST. Empty circles indicate when the prediction skill is statistically significant (*p* < 0.05, see “Methods”).

Figure 1 shows that both the equatorial Atlantic and Pacific regions have very different prediction skill levels across models, as described by the anomaly correlation coefficient (ACC). ATL3 has comparatively lower skill and a large inter-model spread (0.2−0.7 by September). Only a few models maintain skill above persistence during the summer months (JJAS), the period when the Atlantic–Pacific teleconnection is most active. This is therefore a region where seasonal predictions have room for improvement.

By contrast, the Niño3 index is highly predictable, and all systems beat persistence at all forecast times. An important contributor to their high predictive skill is the preconditioning role of local ocean heat content anomalies through a correct initialization^33^. Improvements in initialization, in conjunction with the models’ increasing abilities to represent dynamical aspects of the Bjerknes feedback loop^34^, have been likely contributing to the improvement in ENSO skill in the past decades^35^.

**Fig. 2:**
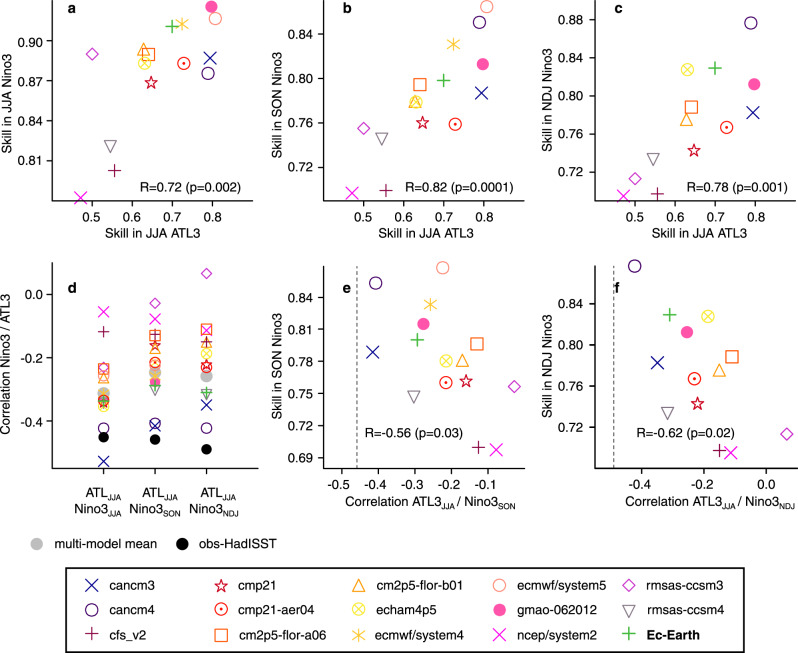
Atlantic/Pacific relationships in the multi-model ensemble. **a**–**c** Scatter plots of prediction skill (in terms of ACC) in JJA ATL3 against the ACC prediction skill of Niño3 SST in JJA, SON, and NDJ, respectively. **d** Strength of the teleconnection in the predictions systems, evaluated by the correlation coefficient of ATL3 SST in JJA and Niño3 SST at different forecast ranges (JJA, SON, and NDJ). The corresponding observed values for HadISST v1.1 are shown with black dots. **e**, **f** Scatter plots of the prediction skill in SON/NDJ Niño3 SST against the strength of the teleconnection between JJA ATL3 SST and SON/NDJ Niño3 SST, respectively. Vertical lines in panels **e** and **f** indicate the strength of the correlations in observations. The period is 1981–2011. In all scatter plots the Pearson’s correlations and corresponding *p* values are shown.

Despite the high level of Niño3 skill, there is an appreciable inter-model spread that grows larger with forecast time (0.5−0.9 by March). This spread could be partly related to the different representation of westerly wind bursts in models, which have been shown in previous studies to limit ENSO forecast quality^36,37^. Part of the spread could also be explained by a different representation of the teleconnection mechanisms with the Atlantic ocean. Figure 2a–c shows that this spread in Niño3 skill in autumn and winter is indeed related to the spread in the preceding summer ATL3 skill. However, this does not necessarily imply causality, as better-performing models could reproduce and predict more accurately the variability in both regions. But it is consistent with previous studies linking summer Atlantic Niños with subsequent ENSO events, and more in particular with those affecting the eastern Pacific region^38^. To shed light on this, we look at the teleconnection and how it is represented. This is done by quantifying in each simulation the correlation coefficient between the summer ATL3 and Niño3 at different lags, to estimate how strong their covariability is and how it evolves in time (Fig. 2d). Also, to evaluate the realism of this teleconnection, we compute the same correlations for the observational dataset HadISST. These correlations are negative, as expected from previous literature linking warm (cold) summer ATL3 phases with the later occurrence of cold (warm) winter ENSO events^9,10^. Their comparison shows that all models underestimate the teleconnection strength (i.e., the magnitude of the negative correlation), with some models showing values close to the observed ones, while in others the correlation is close to zero.

Can this heterogeneous representation in the ATL3-ENSO teleconnection explain part of the previous spread in Niño3 skill? Figure 2e, f supports this hypothesis. For example, SON Niño3 skill is linearly related (*R* = −0.56; *p* < 0.05) with the strength of the teleconnection between ATL3 JJA and Niño3 SON. The same occurs for Niño3 in NDJ (*R* = −0.62; *p* < 0.05) and also for the correlations between ATL3 and Niño3.4 (Supplementary Fig. 1), all consistently suggesting that the higher the ENSO skill is, the stronger (and therefore more realistic) the teleconnection tends to be.

It is important, however, to keep in mind that a more realistic teleconnection alone cannot guarantee high autumn/winter Niño3 skill if the summer ATL3 region is not skillful on its own. This is exemplified by the comparatively low Niño3 skill in rsmas-ccsm4, despite having the third largest ATL3-Niño3 correlation value (thus being the third closest correlation to the observed one). Conversely, a model, like gmao-062012, with really high summer ATL3 skill is outperformed in predicting Niño3 by others with similar or lower ATL3 skill but a more realistic teleconnection, like cancm4 or EC-Earth.

### EC-Earth and sensitivity experiments

To further assess the importance of the equatorial Atlantic and the aforementioned teleconnection on the prediction skill, and corroborate if they are causally linked as we hypothesize from the multi-model analysis, we use EC-Earth to perform a seasonal prediction sensitivity analysis. This comprises three sets of seasonal forecasts covering the period 1981–2018. First, a baseline prediction set labeled “CTR,” the same one already included in the multi-model analysis discussed above (but for the shorter period 1981–2011). A second set, labeled “NUD-VAR,” is performed with an identical configuration to the one from CTR, but with observed SST variability being prescribed in the equatorial Atlantic (between 5°S and 5°N) through the application of a strong nudging^6,12^ (see “Methods”). The model runs freely elsewhere. This experiment is conceived to disentangle the contribution of ATL3 variability to prediction skill in the Pacific. A third set of simulations is additionally produced to investigate the particular role of summer equatorial Atlantic SSTs. This experiment, labeled “NUD-JJAS,” follows the same protocol of “NUD-VAR,” but with SST nudging only being applied from June to September and the model running completely free afterwards.

**Fig. 3:**
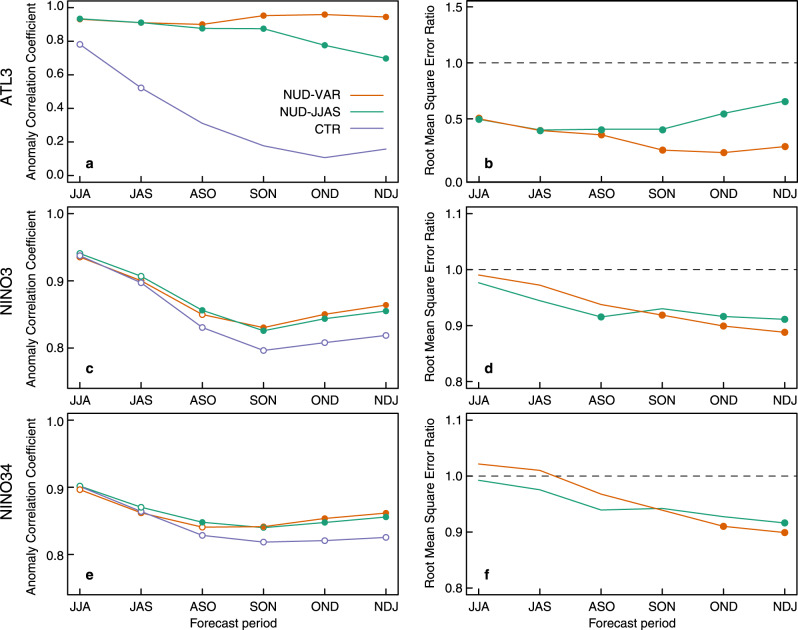
Impact of Tropical Atlantic wind correction on prediction skill. **a** Anomaly correlation coefficient as a function of the forecast period for the ATL3 SST index in the baseline forecast system (CTR) and the two ATL3-nudged ones (NUD-VAR and NUD-JJAS), calculated against HadISST observations. The only difference between the two latter is that in NUD-JJAS the SST nudging over the equatorial Atlantic is only applied during the first 4 forecast months, while in NUD-VAR is applied along the whole forecast. Empty circles indicate when prediction skill is statistically significant (*p* < 0.05, see “Methods”). Full circles indicate when NUD-JJAS or NUD-VAR have significantly higher skill than CTR (*p* < 0.05). **b** Ratio between the root mean square error (RMSE) of ATL3 in the ATL3-nudged forecasts and the corresponding RMSE in the CTR, as a function of forecast time. RMSE ratios <1 denote RMSE reductions in the ATL3-constrained forecasts with respect to CTR. Filled circles indicate when the ratio is significantly different than 1 (*p* < 0.05) according to a two-sided Fisher test. **c**–**f** Same as in panels **a** and **b** but for the Niño3 and Niño3.4 indices. All forecasts metrics are evaluated against HadISST v1.1 observations over the period 1981–2018.

Direct comparison of the three sets of experiments shows that SST nudging boosts, as expected, the prediction skill of ATL3, according to both the ACC and the root mean square error (RMSE) metrics (Fig. 3a, b), with values significantly better in the nudged experiments than in CTR at all forecast times. The RMSE of the forecasts is compared using the ratio between CTR and the nudged experiments, and indicates improvements for values <1. The NUD-JJAS retains high levels of skill for ATL3 beyond September, which might reflect that the signal is more persistent in autumn than in summer (given the faster skill loss in CTR than in NUD-JJAS). In the Pacific basin, the nudged experiments start to show significant improvements in ACC with respect to CTR in early autumn (ASO) and the subsequent months, both for Niño3 and Niño3.4 indices. RMSE improvements are also seen, starting in early autumn in the Niño3 region and in late autumn in the Niño3.4 one. No significant differences are seen between the skill in NUD-VAR and NUD-JJAS, which supports that the decisive contribution of ATL3 to ENSO variability happens in the summer.

**Fig. 4:**
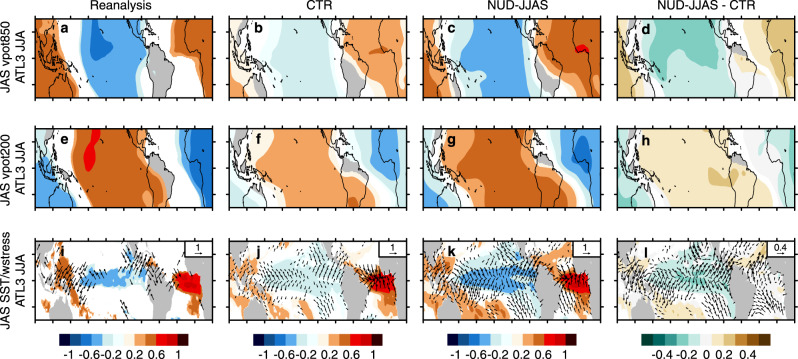
Dynamical description of the ATL3 teleconnection established in JAS. **a**–**c** Correlation pattern between JJA ATL3 index and the JAS velocity potential at 850 hPa for ERA-interim (1st column), CTR (2nd column), and NUD-JJAS (3rd column). Only significant values at a 95% confidence level are shown. **d** Differences between the correlations in panels **c** and **b**. Only significant differences at the 95% confidence level are shown. **e**–**h** The same as in panels **a**–**d** but against the velocity potential at 200 hPa. **i**–**l** As in panels **a**–**d** but for the SSTs (in shaded colors) and the surface winds (in vectors). The period is 1981–2018.

The skill improvements in the nudged experiments are supported by an improvement in the representation of the teleconnection, already visible in JAS as illustrated by the spatial correlations in Fig. 4 and Supplementary Figs. 2–4. For example, for the positive JJA ATL3 phases, observations suggest that one month later (i.e., JAS) there is an increase in convection over the Tropical Atlantic (evidenced by positive correlations in velocity potential at 850 hPa and negative correlations at 200 hPa; Fig. 4a, e) accompanied by enhanced subsidence over the central Tropical Pacific (which shows opposite velocity potential values to the Atlantic). Qualitatively, both CTR and NUD-JJAS are able to represent this response in the Walker circulation (NUD-VAR represents the same overall responses as NUD-JJAS but was excluded from Fig. 4 for conciseness). But while the CTR forecast system clearly underestimates the magnitude of the correlations, these are visibly higher, and therefore closer to the observed ones, in NUD-JJAS (Fig. 4d, h). This improvement manifests clearly at the surface, where the ATL3-nudged experiment develops a more realistic response in SST and winds (Fig. 4i–l). All these quantitative improvements in the representation of the ATL3 teleconnection patterns are also seen at subsequent forecast times (Supplementary Figs. 2–4).

## Discussion

This paper demonstrates the benefits for ENSO predictive skill of having a good representation of ATL3 and its teleconnection over the Tropical Pacific for the study period 1981–2018, in which the teleconnection has been shown to remain active^39^. This has been done by combining a multi-model analysis of 15 state-of-the-art prediction systems from which we have documented substantial inter-model spread in late autumn ENSO prediction skill, and a set of sensitivity experiments that allowed us to single out the Equatorial Atlantic (excluding other neighboring areas) as a key region to understand the inter-model differences.

In the sensitivity experiments, the general improvements that are found after SST corrections are applied on the equatorial Atlantic, which involve a better teleconnection, offer interesting prospects for dynamical seasonal predictions of ENSO. The equatorial Atlantic is a region traditionally affected by long-standing model biases that can deteriorate its predictive skill^32^. Our analysis suggests that efforts to reduce these biases have the potential to also benefit the prediction skill of ENSO, and through it of its most important widespread climate impacts. Interestingly, we have shown that prediction skill improvements can be expected for two different metrics, which represent different aspects of forecast quality. We highlight a potential reduction of up to 10% in the RMSE for autumn/winter ENSO predictions (Fig. 3d, f) when SST variability and mean state are corrected in the equatorial Atlantic. This result implies a better agreement between the amplitude of the observed and predicted anomalies, which also translates into a better prediction of the correct ENSO phase and magnitude^40^.

Recent studies also suggest a prominent influence of the Tropical North Atlantic (TNA) over the tropical Pacific^7,38^, establishing in late winter–early spring and thus coinciding with the onset of ENSO spring predictability barrier. TNA is typically related to central Pacific Niño development through a Gill-type Rossby-wave response^41,42^, while the mechanisms described in our study relate Atlantic Niño to eastern Pacific dynamically developed Niños. Further analyses assessing the impact of the TNA-Pacific teleconnection will have the potential to further expand ENSO predictive capacity over this key season.

## Methods

### Description of the indices

Throughout the manuscript, we use three indices to describe the variability in the tropical basins. The ATL3 index is the area-averaged SST anomaly over 20°W−0 and 3°S−3°N, commonly used to describe the Tropical Atlantic interannual variability, as it covers the area with the largest interannual variance^14^. Similarly, Niño3 and Niño3.4 are the area-averaged SST anomalies over 150°W−90, 5°S−5°N, 170°W−120, and 5°S−5°N, respectively. While both indices are commonly used for measuring ENSO strength variability, they tend to capture events that differ in their spatial structure and magnitude. Niño3.4 is defined over a region that partly covers both the central and east equatorial Pacific, resulting in a blend of the two main ENSO flavors, which are characteristic of both regions^43^. The Niño3 index, on the other hand, is more suited for characterizing the eastern Pacific variability, a region in which ENSO is positively skewed and that usually experiences stronger El Niño than La Niña events^44^.

### Description of the multi-model set of seasonal predictions

The first part of the analysis is based on a selection of seasonal predictions from the North American Multi-Model Ensemble (NMME^45^) and EUROSIP ensembles. Only those initialized on the first of June and with a minimum duration of 6 months were considered to be able to assess the prediction skill of ATL3, ENSO, and their teleconnection from summer through early winter. The period analyzed here is 1981–2011, which is the maximum period with available data for all the models. For each model, we use all members available. We note that our multi-model ensemble of forecast systems was constrained by the study period and the availability of June initialized forecasts, which might have led to an underestimation of the true inter-model ensemble spread. An additional prediction system covering the same period and seasons was produced with EC-Earth for this study. All details are summarized in Supplementary Table 1.

### EC-Earth model configuration

All simulations with EC-Earth were produced with the version 3.1 of EC-Earth^46^. Its atmosphere component is the Integrated Forecasting System (IFS) cycle 36r4, developed in ECMWF (European Center for Medium Range Weather Forecast). IFS is a primitive equation model with fully interactive cloud and radiation physics. Its T255 spectral resolution, used in this study, corresponds to ~0.35° in latitude and longitude. It uses 91 vertical levels (up to 1 Pa) and a time step of 2700 s.

The ocean component is the version 3.3.1 of NEMO (Nucleus for European Modeling of the Ocean)^47^. NEMO uses the so-called ORCA1 configuration, which consists of a tripolar grid with poles over northern North America, Siberia, and Antarctica at a resolution of ~1°, respectively. A higher resolution, by roughly a factor of 3, is achieved close to the equator. Forty-six *z*-coordinate vertical levels are defined together with a partial-step representation of the bottom topography. The vertical grid thickness ranges between 6 m at the surface and 250 m near the ocean bottom. The effects of the subgrid-scale processes (mainly the mesoscale eddies) are represented by an isopycnal mixing/advection parameterization as proposed by Gent and McWilliams^48^, while the vertical mixing is parameterized according to a local turbulent kinetic energy closure scheme^49^. A bottom boundary layer scheme, similar to that in ref. ^50^, is used to improve the representation of dense water spreading.

The Louvain-la-Neuve Sea-Ice Model version 3 (LIM3^51^) is integrated into NEMO, with dynamics based on Beckmann and Döscher^52^ and thermodynamics based on Semtner^53^. The ocean sea-ice component NEMO-LIM3 uses a time step of 3600 s. The atmosphere and ocean sea-ice components of EC-Earth are coupled every 3 h with the Ocean Atmosphere Sea-Ice Soil coupler version 3 (OASIS^54^).

### Protocol for the sensitivity study

Three sets of seasonal hindcasts, also referred to as retrospective forecasts, have been performed with EC-Earth using the Autosubmit workflow manager^55^. The first one, labeled as “CTR,” is the same one used in the multi-model analysis. Its atmospheric component is initialized from ERA-interim reanalysis data^56^, while the ocean component from the ocean reanalysis ORAS4^57^. For each start date, 15 members are produced by applying a small perturbation to the atmospheric initial conditions using the singular vector perturbations method^58^. The hindcasts are initialized every first of June from 1981 to 2018 included, and they are run at the standard resolution ORCA1T255 for 8 months. This set of predictions is used as the reference against which the two perturbed predictions, described below, are compared.

The first set of perturbed predictions, labeled “NUD-VAR,” follows an identical protocol to CTR, with the additional feature that ORAS4 SST values are prescribed through nudging between 5° N and 5° S in the Atlantic basin during the whole length of the forecasts. This is done by applying a strong nudging coefficient of −200 W/m^2^/K, which constrains tightly the simulated SSTs to the observations (as evidence in Fig. 3a).

A second set of perturbed predictions (NUD-JJAS) was additionally performed, following the same protocol of NUD-VAR but nudging the Tropical Atlantic SSTs only during the first 4 forecast months, and letting the model run free after September.

### Forecast verification

The retrospective forecasts have been evaluated against a number of different products, depending on the variable. These products are also used for assessing the ability of the models to reproduce different aspects of the teleconnection mechanism through linear regressions. For SST we use the monthly gridded observational dataset HadISST v1.1 from UK Met Office^59^. For surface winds, we use the ERA-Interim reanalysis^60^. For consistency among the verification products, our reference velocity potential at different heights is computed from ERA-Interim velocity fields using the corresponding functions from the Climate Data Operators v1.6.3^61^. Forecast evaluation of the multi-model ensemble is done using seasonal averages.

### Tools and metrics for the statistical analysis and significance assessment

The statistical analysis and the significance assessment have been performed with two R-packages, s2dverication^62^ and SpecsVerification (https://cran.r-project.org/web/packages/SpecsVerification/). ACC and RMSE metrics are calculated from ensemble means. In the correlation maps that involve simulations, the ensemble members are concatenated into a single time series prior to the computation. This is done because ensemble means tend to filter out part of the noise, which leads to substantially higher correlation values than for observations^63^. By concatenating the members, correlations do not suffer from this effect, but usually yield more significant results due to the increase in sample size. The significance levels for the ACC skill score and the correlation coefficients are calculated with a Student’s *t* test. For evaluating the significance in the differences between the correlations patterns of CTR and NUD-VAR/NUD-JJAS, we use a bootstrap method with a sample of 1000, making use of multiple computing cores to speed up the calculation with the function multiApply (https://CRAN.R-project.org/package=multiApply) of the s2dverification R-package. For evaluating the differences between the ACC skill scores, we use statistical tests based on a power analysis^64^, described in ref. ^65^, and implemented in the function CorrDiff of the SpecsVerification R-package. The advantage of this method over other commonly used ones (e.g., the test based on Fisher transformation) is that it has higher statistical power (the probability of correctly detecting improvements in the skill) when the two forecasts are strongly correlated with each other, which is the case in the ENSO region.

To estimate if the RMSE values in CTR and the perturbed experiments are significantly different, we use a two-sided Fisher test, considering the null hypothesis that the RMSE ratio between them is equal to 1.

### Sensitivity of the results to the methodological choices

Different sensitivity tests have been performed to evaluate the robustness of our results, all of them confirming the main conclusions of this study: (1) models with better ATL3 skill tend to predict better ENSO, (2) models with a better ATL3/ENSO teleconnection also show enhanced ENSO skill, and (3) improving the representation of Tropical Atlantic SSTs (both in mean state and variability) have a beneficial impact on both the ATL3/ENSO teleconnection and ENSO skill. These sensitivity tests include considering shorter periods for the multi-model analysis, splitting the analysis into periods of predominantly positive/negative AMV phases, changing the reference observational dataset to evaluate the forecast skill (from HadISST to ERSST), and considering a different experimental approach based on prescribing surface wind stress to constrain the Tropical Atlantic SSTs.

## Supplementary information

Supplementary information

## Data Availability

NMME data have been downloaded from the IRI database (http://iridl.ldeo.columbia.edu/SOURCES/.Models/.NMME/). EUROSIP and ERA-interim data have been downloaded from the MARS computing facility of ECMWF (https://www.ecmwf.int/en/forecasts/). Data from EC-Earth predictions are available on request from the corresponding author (E.E.). HadISST v1.1 and ERSSTv4 are publicly available at: https://www.metoffice.gov.uk/hadobs/hadisst/data/ and https://www1.ncdc.noaa.gov/pub/data/.

## Code availability

The code developed in this study is available in the following GitHub repository: https://github.com/eexarchou/NCOMMS-19-31255-T. It is written using the “R” program language and the NCAR Command Language (NCL)^66^. We have used the CRAN packages s2dverification^62^ and SpecsVerification.
