# Supplementary material for: Impact of equatorial Atlantic variability on ENSO predictive skill
Source: Nat Commun. 2021 Mar 12;12:1612. doi: 10.1038/s41467-021-21857-2 (PMC7954793; doi:10.1038/s41467-021-21857-2)
Supplement: Supplementary file 1 — Supplementary information [file 41467_2021_21857_MOESM1_ESM.pdf]

## A Supplementary Information

**Supplementary Table 1.** Seasonal predictions available in the EUROSIP and NMME[1] multi-model ensemble forecasts systems. In **bold** are predictions that are used in this study. EC-Earth predictions are performed locally at BSC. Predictions not used in this study did not have available data for the variables or period used in this study at the time of writing.

| Model | Origin | Ensemble size | Length | Period |
|---|---|---|---|---|
| **NCEP (system2)** | EUROSIP | 24 | 9 mon | 1982 – 2010 |
| ECMWF(system3) | —"— | 41 | 7 mon | 1981 – 2005 |
| **ECMWF (system4)** | —"— | 15 | 7 mon | 1981 – 2010 |
| **ECMWF (system5)** | —"— | 25 | 6 mon | 1981 – 2016 |
| GLOSEA5 | —"— | 8 | 6 mon | 1992 – 2011 |
| MeteoFrance (system3) | —"— | 41 | 7 mon | 1981 – 2005 |
| MeteoFrance (system4) | —"— | 51 | 7 mon | 1991 – 2010 |
| MeteoFrance (system5) | —"— | 15 | 7 mon | 1991 – 2014 |
| **cfs_v2** | NMME | 24 | 10 mon | 1982 – 2011 |
| **cancm4** | —"— | 10 | 10 mon | 1981 – 2011 |
| **cancm3** | —"— | 10 | 10 mon | 1981 – 2011 |
| **cm2p5-flor-a06** | —"— | 12 | 10 mon | 1980 – 2011 |
| **cm2p5-flor-b01** | —"— | 12 | 10 mon | 1980 – 2011 |
| **rsmas-ccsm4** | —"— | 10 | 10 mon | 1982 – 2011 |
| **rsmas-ccsm3** | —"— | 6 | 10 mon | 1982 – 2011 |
| **cm2p1** | —"— | 10 | 10 mon | 1982 – 2011 |
| **cm2p1-aer04** | —"— | 10 | 10 mon | 1982 – 2011 |
| **echam4p5** | —"— | 12 | 8 mon | 1982 – 2011 |
| **gmao-062012** | —"— | 12 | 9 mon | 1981 – 2011 |
| gmao | —"— | NA | NA | NA |
| cesm | —"— | NA | NA | NA |
| **EC-Earth** | BSC | 15 | 8 mon | 1981 – 2018 |

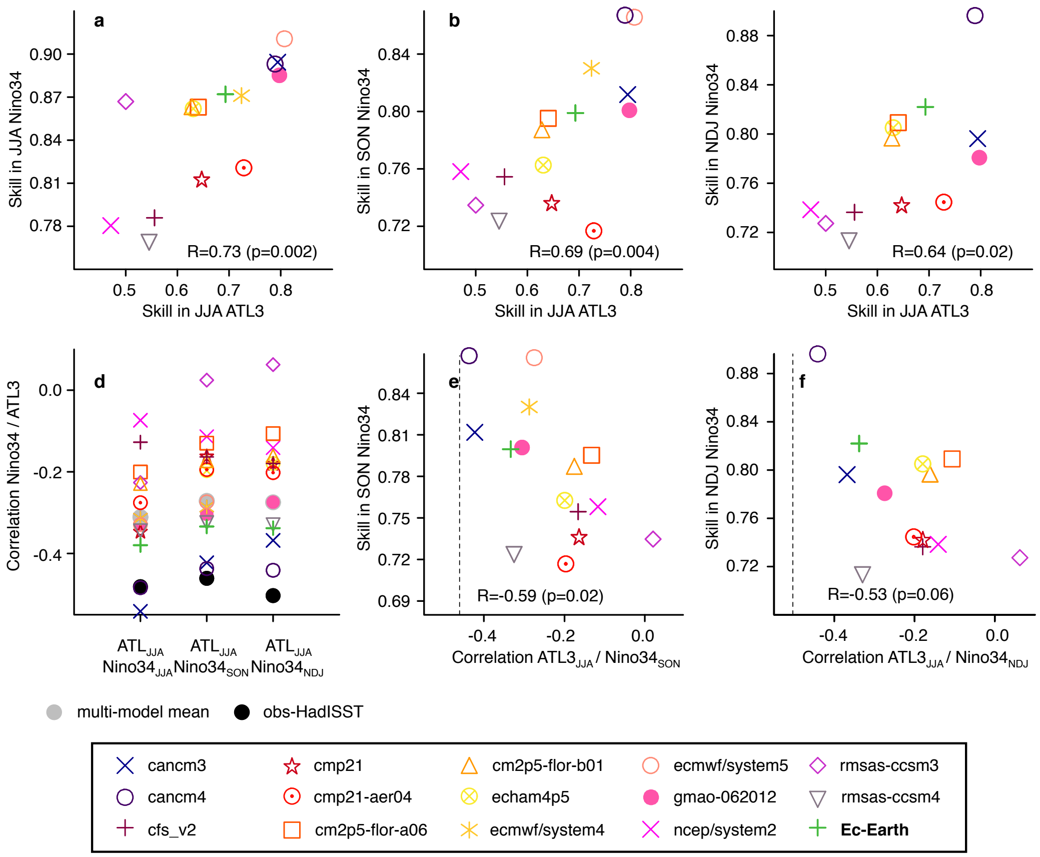


**Supplementary Figure 1.** As in Fig 2 but for the Niño3.4 index instead.

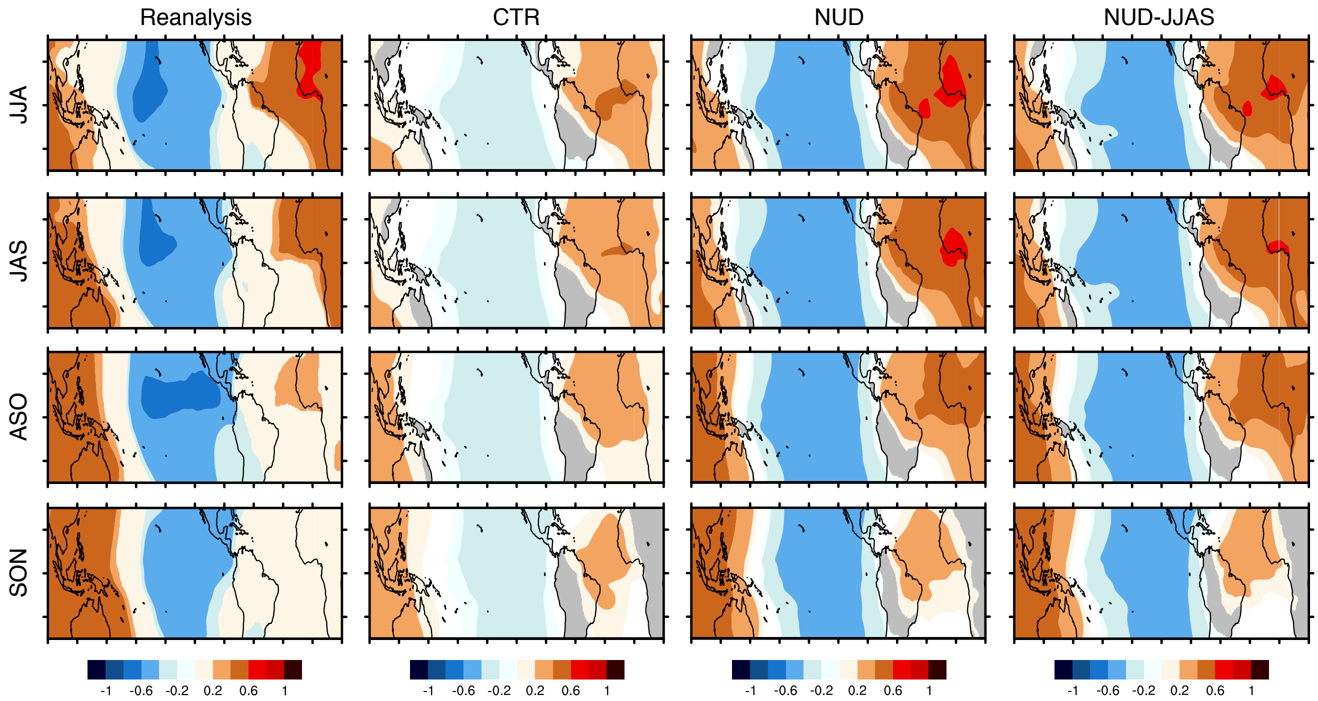


**Supplementary Figure 2.** As in Fig 4i-l but for the velocity potential at 850 hPa for the 3-month periods: JJA, JAS, ASO and SON.

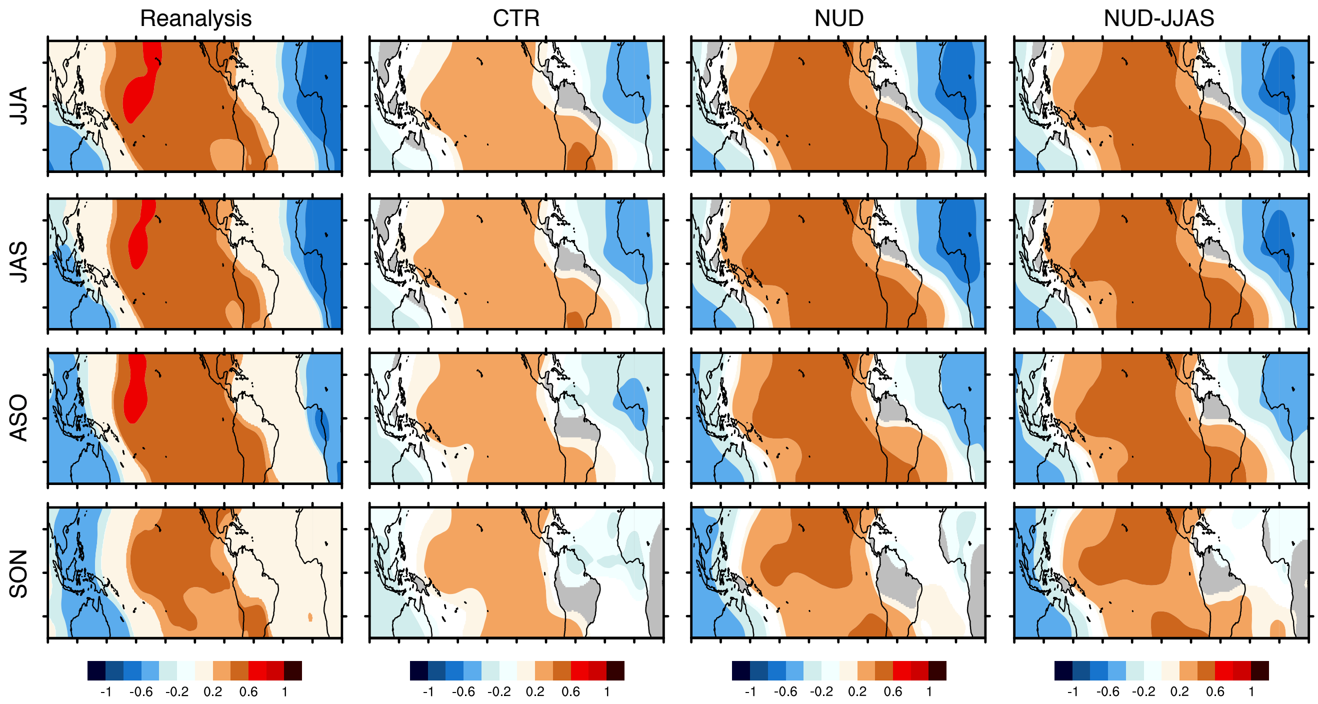


**Supplementary Figure 3.** As in Supplementary Fig 2 but for the velocity potential at 200 hPa for the 3-month periods: JJA, JAS, ASO and SON.

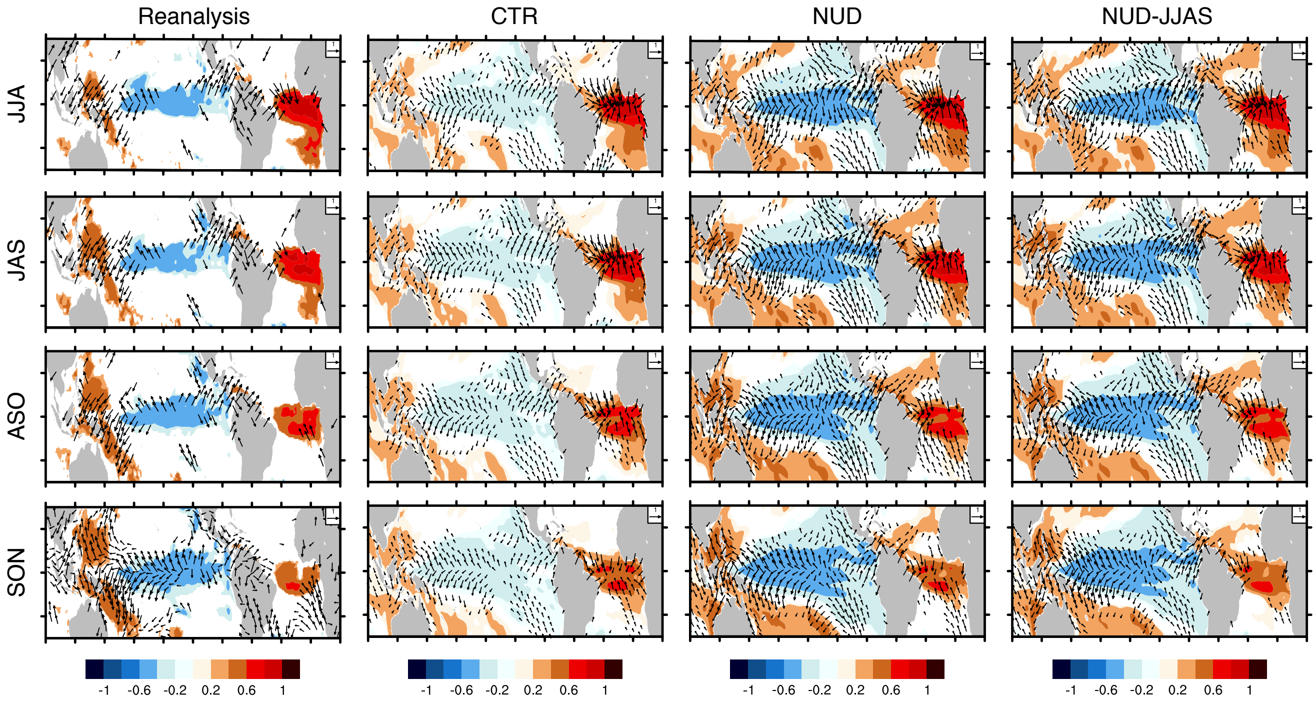


**Supplementary Figure 4.** As in Supplementary Fig 2 but for SSTs from the 3-month periods: JJA, JAS, ASO and SON.

## References


1. Kirtman, B. P. *et al.* The North American multimodel ensemble: phase-1 seasonal-to-interannual prediction; phase-2 toward developing intraseasonal prediction. *Bull. Am. Meteorol. Soc.* **95**, 585–601 (2014).
